# An *In Vitro* Comparative Study of the Adaptation and Sealing Ability of Two Carrier-Based Root Canal Obturators

**DOI:** 10.1155/2013/532023

**Published:** 2013-05-09

**Authors:** Ahmed Alkahtani, Sara Al-Subait, Sukumaran Anil

**Affiliations:** ^1^Department of Restorative Dental Sciences, College of Dentistry, King Saud University, P.O. Box 300353, Riyadh 11372, Saudi Arabia; ^2^Dental Caries Research Chair, Department of Restorative Dental Sciences, College of Dentistry, King Saud University, Saudi Arabia; ^3^Department of Periodontics and Community Dentistry, College of Dentistry, King Saud University, P.O. Box 60169, Riyadh 11545, Saudi Arabia

## Abstract

The study was done to assess the sealing ability and adaptation of RealSeal 1, and to compare it with Thermafil. 65 single-rooted extracted teeth were selected and root canal treatment was performed. Root canals were obturated with RealSeal 1 or Thermafil. A double chamber bacterial leakage model using *E. faecalis* was developed to assess the sealing ability. Samples were monitored daily for 60 days. After the bacterial leakage test, samples were embedded in resin and sectioned horizontally at 2 and 4 mm from the apical foramen. Specimens were examined under scanning electron microscope and digitally photographed. AutoCAD software was used to measure the gap between the canal surface and obturation material. Results were statistically analyzed using nonparametric Kaplan-Meier survival analysis for the bacterial leakage and *t*-test to compare the means of gap in RealSeal 1 and Thermafil at 2 and 4 mm. There was no significant difference between the RealSeal 1 and Thermafil with respect to leakage over time. At 2 mm and 4 mm, RealSeal 1 had significantly more gaps than Thermafil. From the observations it can be concluded that RealSeal 1 and Thermafil have comparable performance in terms of adaptation and sealing ability.

## 1. Introduction

Complete obturation of the root canal with filling material and creation of a hermetic apical seal are the goals of endodontic treatment. It has been shown that approximately 60% of endodontic failures are due to inadequate obturation of the root canal system. Hence it is important to use materials which are able to create a hermetic seal between the root canal system and periapical tissue [1]. In order to prevent re-infection of the root canal via leakage of microorganisms and their byproducts, the sealing ability, biocompatibility [2, 3], and antimicrobial effect of root canal filling materials are important factors to be considered [4]. Attempts were made to develop new filling materials and techniques to increase the quality of the canal seal.

The most widely used root canal filling material is gutta-percha due to its inertness, plasticity, and solvent solubility [5]. Gutta-percha does not bond to root dentin and therefore must be used in conjunction with a sealer cement [6]. The Thermafil technique involves the obturation of the root canal with heated gutta-percha on a plastic carrier. This carrier-guided gutta-percha technique is easier to reduce the sealer component, showing less leakage *in vitro* compared with the lateral compaction technique [7].

Resilon carrier obturation system (RealSeal 1; SybronEndo, CA, USA) has been introduced into the market, combined with a self-etch sealer (RealSeal-1, SybronEndo, Orange, CA, USA). The sealer components are methacrylate monomers, partially containing carboxylic acid groups, fillers of calcium phosphate, Ca-Al-F-silicate (glass ionomer powder), silanated barium borosilicate glass, and radiopaque fillers. The carrier is a polysulfone-containing polymer with radiopaque filler, and the surrounding Resilon contains polycaprolactone and polyolefin polymers loaded with fillers, such as bioactive glass, bismuth oxychloride, and barium sulfate [8].

Several methods have been used for evaluating the adaptability and sealing ability of root canal filling materials, such as dye penetration test, fluid filtration methods, radioactive isotope studies, electrochemical leakage tests, scanning Electron microscopic (SEM) analysis, and bacterial penetration test. Scanning electron microscopic is used to evaluate the sealing ability and addictiveness of the root canal filling materials. Although the use of dyes, radioisotopes, fluid filtration, bacteria, and endotoxin penetration techniques has been used to evaluate the seal of endodontic material, the bacterial leakage model has been advocated as a more clinically relevant model [9]. Studies have shown that the thermoplasticized Resilon can flow into grooves and depressions in a split tooth model or in artificial lateral canals in a similar fashion to gutta-percha [10, 11]. Several claims have been made by the RealSeal system; for example, it bonds to the core eliminating a potential pathway for bacterial leakage and the RealSeal-1 is injection molded, ensuring that the core is centrally placed in the outer Resilon.

Even though several claims were made by the new system, only limited research has been done to compare the RealSeal 1 to the carrier-based Thermafil system in terms of bacterial leakage and the sealing ability [12, 13]. The objective of the study was to compare microleakage and the sealing ability in teeth filled with RealSeal 1 versus Thermafil using *Enterococcus faecalis *as a bacterial marker. The adaptability of these two materials to the root canal wall was assessed using scanning electron microscopy.

## 2. Materials and Methods

### 2.1. Collection of Teeth

A total of sixty-five single-rooted extracted teeth were used in this study. All teeth selected for the study met the following criteria:

- teeth with one, noncalcified canal, which was confirmed by radiographs taken from buccolingual and mesiodistal views;
- root canal curvature was less than 30 degrees, according to Schneider's method [14];
- teeth had mature apices, and roots were free from cracks, resorption, caries, and restoration.

All selected teeth were stored in 0.5% sodium hypochlorite (NaOCl) in room temperature to prevent growth of bacteria.

### 2.2. Root Canal Preparation and Filling

The teeth were decoronated to give approximately 14 mm of root length from the coronal surface to the apex of the root using a rotating diamond saw under water cooling. The working length was determined by placing size 10 K-file (Maillefer, Ballaigues, Switzerland) in the canal until it was visible at the apical foramen and subtracting 1 mm from this measurement. The canals were prepared with Gates Glidden Burs (Maillefer, Dentsply, Switzerland) sizes 4, 3, and 2 for the coronal third. Glide path was established by hand filing with K-file sizes 15, 20, and 25 to full working length. Instrumentation was completed with a high torque motor at 300 rpm and 0.06 Taper Profile nickel-titanium rotary instruments (Dentsply Tulsa Dental, Tulsa, Oklahoma, USA) up to size 40 by crown-down technique. RC-Prep root canal lubricant (Premier Dental Products, King of Prussia, PA, USA) was used with rotary files. Instruments were replaced after every five-root canal preparation or if any sign of deformation was observed.

Canals were irrigated with 2.25% NaOCl after each change of instrument with a syringe and a 30 gauge Stropko NiTi Flexi-Tip endodontic irrigating needle (SybronEndo, Crop, Orange, CA, USA). Apical patency was maintained by passing size 10 K-file after each instrument 1 mm beyond the working length. After completion of instrumentation, the smear layer was removed with 10 mL of 17% Ethylenediaminetetraacetic acid (EDTA) (Pulpdent, Watertown, USA) applied over a period of 1 minute, followed by 10 mL 2.25% NaOCl. The canals were then flushed with 30 mL of sterile saline to remove the residual irrigant and dried with sterile paper points (Dentsply Maillefer, Tulsa, Oklahoma, USA). The same operator carried out the instrumentation. After root canal preparation, roots were randomly assigned to four groups: two experimental groups of twenty-five roots each, and fifteen roots assigned to the positive and negative control groups.

### 2.3. Group 1

#### 2.3.1. RealSeal 1 (*n* = 25)

A size verifier that fitted passively in the canal was selected. The corresponding size obturator that matched the size verifier was chosen. A thin film of RealSeal 1 self-etching sealer was placed along the walls of the entire canals via size 40 K-file as the manufacturer's instructions. Each obturator was then heated in a RealSeal 1 Oven and inserted into the prepared canal within 6 seconds of removing it from the oven in accordance to the manufacturer's instructions. The handle of each obturator was stabilized while the carrier was sectioned at the orifice level using a bur. Excess filling material surrounding the carrier was compacted apically by using Buchanan pluggers (SybronEndo, Crop, Orange, CA, USA). The coronal surface of the RealSeal 1 was light cured (Elipar Highlight, 3M, ESPE, St. Paul, MN, USA) for 40 seconds to create an immediate coronal seal about 0.5 to 1 mm deep.

### 2.4. Group 2

#### 2.4.1. Thermafil (*n* = 25)

In order to determine the size of the Thermafil obturator, a size verifier was used. Size 40 K-file was used to apply thin coating of Topseal root canal cement (Maillefer, Ballaigues, Switzerland) over the entire working length of the root canal walls. Each obturator was heated in a ThermaPrep Plus Oven until an audible signal indicated that the obturator was ready for placement. It was then inserted into the prepared root canal with a slow, firm, and continuous movement in apical direction. The handle of each obturator was stabilized and a bur was used to section the carrier at the orifice level, following manufacturer's guidelines. Excess gutta-percha surrounding the carrier was compacted apically by using Buchanan pluggers.

#### 2.4.2. Positive Control (*n* = 5)

The prepared root canals were not obturated. This group was included to demonstrate bacterial leakage through the entire length of the canal.

#### 2.4.3. Negative Control (*n* = 10)

Canals were filled in the same manner as in group 1, with RealSeal 1 (*n* = 5), and group 2, with Thermafil (*n* = 5). Root surfaces were covered with two coats of colored nail varnish (Bourjois, Paris) except for coronal access.

After canal preparation, roots were stored in gauze that was dampened with saline and enclosed in sealed tubes for two weeks at 37°C to allow the sealer to set. The external surfaces of all roots in the experimental and positive control groups were covered with two coats of colored nail varnish from the coronal edge to 2 mm short of the apex, to prevent side leakage through the dentinal tubules. 

### 2.5. Leakage Apparatus Preparation

The bacterial leakage model consists of an upper and a lower chamber as described by Torabinejad et al. [15] with some modifications. A 60 mL container was modified to create a dual-chamber device. A hole was created in the center of each plastic cap for the fitting of the root. 5 mm of the tapered end of a 1.5 mL Eppendorf plastic tube (Eppendorf-Elkay, Shrewsbury, MA, USA) was cut off and the obturated roots were inserted in the tubes with 4 to 5 mm of the roots protruded through the end. The junction between the tube and root was sealed with cyanoacrylate glue (UHU GmbH & Co. KG, Bühl, Germany) and two additional layers of colored nail varnish was applied to prevent leakage of the connection. These roots and the prepared Eppendorf tubes assemblies were snugly fit into the plastic cap of the container. Each assembly was sterilized by a 12-hour cycle in an ethylene oxide gas sterilizer (3M Steri-Vac Gas Sterilizer, 3M Health Care, Minnesota, USA). After sterilization and degassing, all procedures were carried out in biological safety cabinet. Sterile Brain Heart Infusion Broth (BHIB, Oxoid Ltd., UK) was aseptically placed into the container to a level of nearly 2 mm above the root end. The assemblies were incubated at 37°C for 24 hours to ensure no contamination occurred before inoculation. Any sign of turbidity in the medium would indicate bacterial contamination and the system would have to be discarded.

*Enterococcus faecalis*, obtained from American Type Culture Collection (ATCC) 29212, was cultured at 37°C for 24 hours in BHIB. 0.5 mL aliquots of the *E. faecalis* culture were used to inoculate the upper chambers using a micropipette. To ensure the viability of the bacteria throughout the experiment, the inoculum of *E. faecalis* in the top chambers was changed with fresh inoculum twice weekly under a laminar airflow hood. The assemblies were incubated at 37°C for 60 days. During this period, the medium in each container was monitored daily for signs of turbidity, which indicates leakage had occurred throughout the 14 mm length of the filled root canal. If turbidity occurred in the lower chamber, the seal was broken; a sample of the turbid fluid was taken and subjected to gram staining and light microscopy (Leitz, Switzerland) to assess the colony morphology at 1000x magnification. When the presence of *E. faecalis *was confirmed, the time from inoculation to turbidity was observed and recorded. 

### 2.6. Specimen Embedding and Sectioning

After the bacterial leakage test, samples were removed from the Leakage Apparatus. A rubber container was used to embed the samples in a clear orthodontic resin (Technosin, Protechno, Girona, Spain). After mixing the resin according to the manufacturer instructions, the roots were embedded and left for 24 hours for curing. Specimens were then removed from the apparatus, and resin apical to the root end was ground until first contact with the root tip. Each sample was sectioned horizontally 2 and 4 mm from the apical foramen using Isomet low speed saw (Isomet; Buehler Ltd., Lake Bluff, NY, USA) running at 5,000 rpm with a diamond disk (127 mm × 0.5 mm) and continuous water irrigation in order to prevent frictional heat, which may result in smearing of the sample. Samples were oriented so that sectioning was perpendicular to their long axis. Each slice was marked to distinguish the coronal from the apical side. Debris was removed and sections were smoothed with 600-grit wet silicon carbide sandpaper (Leco, St. Joseph, MI, USA) before microscopic examination.

### 2.7. Microscopic Evaluation

The sections prepared for SEM examination were air-dried and sputter-coated with gold using fine-coat ion sputter JFC-1100, JEOL Ltd., Tokyo, Japan). The specimens were examined under scanning electron microscope (SEM) (JEOL JSM-6360LV, Tokyo, Japan) and digitally photographed at 100x magnification. AutoCAD software was used for image analysis. Through this software, the presence of any gap between the canal surface and obturation was measured and calculated as a percentage from the total canal periphery.

### 2.8. Statistical Analysis

For the bacterial microleakage test, data are presented showing the mean and median leakage times in days. Maximum and minimum values are also presented. Nonparametric Kaplan-Meier survival analysis was used to estimate the mean and median for survival time and to compare the survival curve patterns between RealSeal 1 and Thermafil. Leakage was statistically compared between the two groups using the Log Rank test at level of significance set at 0.05. For the SEM image analysis, Kolmogorov-Smirnov test for normality revealed a normal data distribution and Levene's test revealed homogeneity of variances; hence *t*-test was used to statistically compare the means of RealSeal 1 and Thermafil at 2 and 4 mm with the significance level set at 0.05. SPSS statistical software (version 16; SPSS Inc., Chicago, IL, USA) was used for statistical analysis of the data.

## 3. Results

### 3.1. Bacterial Microleakage

**Figure 1:**
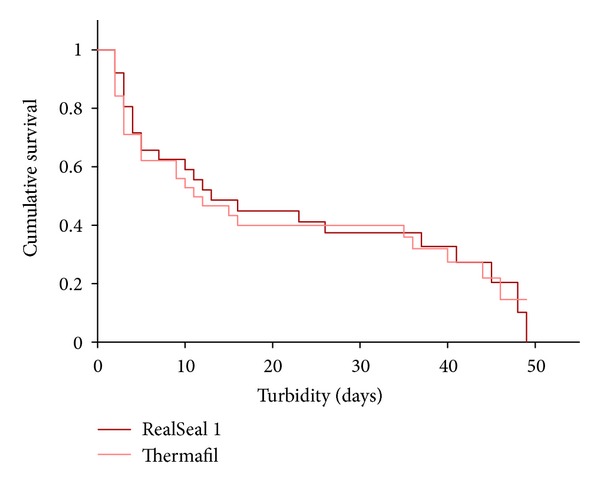
Cumulative survival rates of samples obturated with RealSeal 1 and Thermafil.

The results obtained during the bacterial microleakage test are presented in Table 1. The positive control group specimens leaked within the first 48 hours. None of the negative control group leaked during the whole observation period. The experiment was terminated at the 49th day because all specimens in the experimental groups showed microleakage within 49 days. RealSeal 1 specimens have a median leakage time of 7 days (minimum 2 days and maximum 49 days). Specimens in Thermafil group have a median of 5 days (minimum 2 days and maximum 46 days). The estimated mean day of leakage to occur in the RealSeal 1 group was 15 days and 12 days in the Thermafil group (Table 1 and Figure 1). Statistical analysis using Log Rank test showed no significant difference between the RealSeal 1 and Thermafil with respect to leakage over time (*P* = 0.308).

### 3.2. Scanning Electron Image Analysis

**Figure 2:**
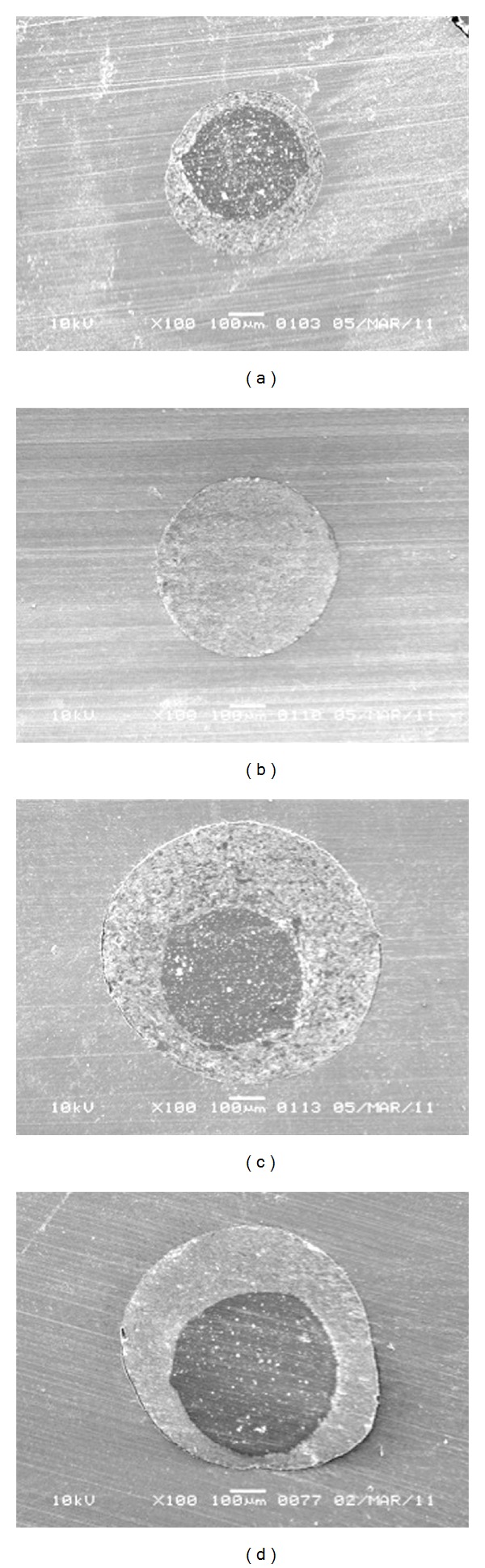
Scanning electron microscopic image of the cross-section of the canal at 2 and 4 mm away from the apex (100x). (a) 2 mm filled with RealSeal 1, (b) 2 mm filled with Thermafil, (c) 4 mm filled with RealSeal 1, and (d) 4 mm filled with Thermafil.

**Figure 3:**
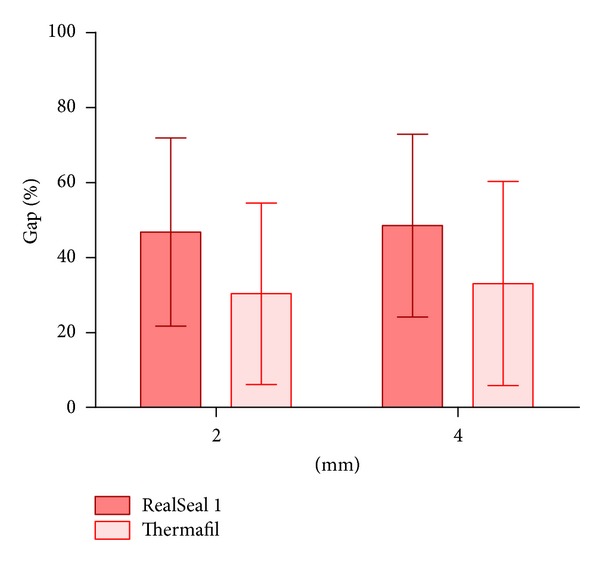
Showing the mean percentage of gap observed in RealSeal 1 and Thermafil at 2 mm and 4 mm sections away from the root apex.

Scanning electron microscopic image of the cross-section of the canal at 2 and 4 mm away from the root apex is shown in Figure 2. The mean percentage gaps observed in both groups are depicted in Table 2 and Figure 3. Independent *t*-test comparing the gaps in RealSeal 1 with Thermafil at 2 mm showed the mean of gap between the obturation material and canal wall in RealSeal 1 = 46.78 (SD = 25.1) and the mean of gap in Thermafil = 30.35 (SD = 24.2). RealSeal 1 at 2 mm had significantly more gaps than Thermafil group (*P* = 0.012). At 4 mm, the mean of gap in RealSeal 1 = 48.49 (SD = 24.4), in Thermafil = 33.03 (SD = 27.2). Statistical analysis showed more gaps between the obturation material and canal wall in RealSeal 1 than in Thermafil at 4 mm (*P* = 0.024).

## 4. Discussion

Root canal obturation is the critical determinant of the success or failure of the endodontic treatment. Inability to completely obliterate the irregularities of root canal spaces with the filling material and adequately seal the apical foramen accounts for nearly 60% of root canal failures. Studies have revealed that inadequate flow of gutta-percha and its inability to adhere to dentinal walls as major determinant leading to an insufficient seal.

Several new sealers and root canal obturation systems are being introduced to improve the sealing abilities of root canal materials. *In vitro* and animal studies established that none of these materials were able to establish all the requirements for a perfect root canal seal [16]. Studies have shown that teeth obturated with Thermafil showed bacterial leakage [17–19]. Therefore, there has been an effort to develop new carrier-based obturation material. RealSeal 1, according to the manufacturer, bonds to both the obturating material and the root canal walls to provide a better seal of the root canal by creating a “monoblock”. 

Leakage studies on the sealing properties of endodontic materials constitute an important area of research [20]. Different methods have been used to demonstrate the sealing ability of materials. A bacterial leakage model may provide a more accurate indicator of clinical applications; therefore, in the present study an *Enterococcus faecalis* leakage model was used to compare the sealing ability of RealSeal 1 to that of Thermafil. *Enterococci* are gram-positive cocci that can occur singly, in pairs, or as short chains. They are facultative anaerobes, possessing the ability to grow in the presence or absence of oxygen [21]. Of the *Enterococcus *species, *Enterococcus faecalis *is the most commonly detected species from oral infections [22]. *E. faecalis *is associated with different forms of periradicular disease and is more likely to be found in cases of persistent infections than in primary endodontic infections [22]. 

During the bacterial microleakage experiment, all positive control specimens leaked within 24 to 48 hour, indicating the ability of *E. faecalis* to penetrate the prepared root canals. Of the negative control specimens none leaked within the experimental period, indicating that the seal created between the two chambers of the systems was efficient. Some samples of the experimental groups leaked at the first 48 hours of observation. These are, in accordance with other *E. faecalis* microleakage, studies that showed leakage after the first day of observation when comparing Resilon and gutta-percha [23, 24]. Results from the present study failed to show statistically significant differences in terms of bacterial leakage of *E. faecalis* in teeth filled with RealSeal 1 compared with Thermafil. Resilon is a polycaprolactone-based thermoplastic root-filling material. Tay et al. [25] found alkaline hydrolysis to occur in the polycaprolactone-based Resilon. This causes biodegradation of Resilon under the attack of hydrolytic ester bond-cleaving enzymes that may exist as a component of the salivary enzymes or as extracellular enzymes from endodontically relevant microbes. Thus, in the event of coronal leakage, Resilon is exposed to different microbes that will result in biodegradation of the filling material and compromise the sealing of endodontic treatment. The dimensional change of Epiphany SE sealer also affects the bacterial leakage of RealSeal system [26]. Another concern with the use of carrier-based root-filling material is the possibility of disrupting the seal during removal of the handle of RealSeal 1 and Thermafil, once the filling material cooled down. In a study, Testarelli et al. [13] demonstrated significantly better sealing in RealSeal 1 than Thermafil which is in contrast to the observations of the current study. This could be attributed to the variations in the methodology as well as the short-term (24 hour) sealing ability assessment. Paque and Sirtes [27] found no significant difference in leakage between Resilon/Epiphany or gutta-percha/AH Plus, in lateral or vertical compaction immediately after obturation. However after 16 months Resilon leaked significantly more than gutta-percha.

Although the bacterial model system used in this study has the advantage of being more clinically relevant, it fails to simulate the oral environment. Also the quantitative measurement of the bacteria was not possible; hence SEM was used as an additional parameter to assess the quality of the filling. Gaps presented between the filling material and root canal might provide a pathway for microorganisms and their by-products to the periapical tissues. Therefore the assessment of these gaps might have a clinical relevance. The quality of root canal filling in apical third is important because of the root canal ramifications in this region. In the present study the adaptation of Thermafil and RealSeal 1 to the canal walls at 2 and 4 mm was assessed using SEM and the gaps were quantified using AutoCAD image analysis software. Results of the present study showed significantly inferior adaptation of RealSeal 1 to the canal walls when compared to Thermafil at 2 and 4 mm. 

Tay et al. [25] evaluated the quality of apical seal achieved with the Resilon/Epiphany and they concluded that it was not superior to AH Plus gutta-percha combination. They found weak link in gutta-percha filled canals between gutta-percha and sealer while in Resilon-filled root canals the weak link was in the sealer-dentin interface. It was concluded that the gap in RealSeal system could be related to the polymerization shrinkage of the Epiphany sealer [25]. Another factor that is associated with polymerization of methacrylate-based resins that could affect the sealing of RealSeal 1 is shrinkage stresses. The configuration factor (C factor) is the ratio of the bonded to the unbonded surface areas. During polymerization, the unbonded surface can move and flow to relief shrinkage stresses. A major problem in a long narrow root canal is that the unbonded surface area is small, that is, lack of relief of shrinkage stresses that might result in the debonding of one or more of the bonded areas [28]. It has been stated that bonding of adhesive root-filling materials to root canals results in exceedingly high C factor when compared to indirect intracoronal restorations with a similar resin film thickness [28].

In the present study, sections were taken at 2 mm and 4 mm away from the apex to get gap-free sections. It was reported that a high percentage of oval canals were observed at 5 mm away from apex which cannot be completely obturated [29–31]. The presence of gap in both materials can be related to the carrier-based technique. The carrier-based filling technique does not employ compaction in the apical third while the material is cooling down to compensate for the material shrinkage, which is considered a limitation of this obturation technique [25, 28]. Furthermore, the presence of gaps in most of the specimens in both experimental groups can explain the bacterial leakage of *E. faecalis* in both materials.

## 5. Conclusion

Based on the results of the study it can be concluded that RealSeal 1 and Thermafil have comparable performance in terms of adaptation and sealing ability. Further randomized clinical trials (RCTs) may be carried out to substantiate the observations of the *in vitro* study.

## Figures and Tables

**Table 1:** Mean number of days from bacterial inoculation to microleakage of teeth filled with RealSeal 1 and Thermafil.

Material	Number of days			
	Mean ± SEM	Median	Minimum	Maximum
RealSeal 1	15.12 ± 3.23	7	2	49
Thermafil	12.56 ± 2.96	5	2	46

**Table 2:** The mean percentage gap observed in RealSeal 1 and Thermafil at 2 mm and 4 mm sections away from the root apex.

	Mean gap—percentage (%) from the total canal	
	periphery	
Material	2 mm	4 mm
	Mean ± SD	Mean ± SD
RealSeal 1	46.78 ± 25.1	48.49 ± 24.4
Thermafil	30.35 ± 24.2	33.03 ± 27.2
